# Annual of the Universal Medical Sciences

**Published:** 1889-10

**Authors:** 


**Annual of the Universal Medical Sciences—A Yearly Report of the Progress of the General Sanitary Sciences throughout the World.** Edited by CHARLES E. SAJOUS, M. D., Lecturer on Laryngology and Rhinology, in Jefferson Medical College, Philadelphia, etc., and seventy associate editors, assisted by over 200 corresponding editors, collaborators and correspondents. Illustrated with chromo-lithographs, engravings and maps. Comprised in four standard octavo volumes of over 400 pages each. F. A. Davis, publisher: Philadelphia, New York and London. 1889.

The title above given indicates the scope of this unique and comprehensive work. When the series of 1888 appeared, we expressed our opinion of the value of this annual, according to it the fullest praise, and, upon a more complete examination, we see no reason to regret the stand then taken, or to recall the words we then wrote. On the contrary, we regard it of inestimable value as a work of reference, and we should find it difficult to dispense with it.

This new series of 1889 more than confirms our previous judgment of this class of reference books, and we desire to record ourselves in unqualified approbation thereof. Several new features have been introduced, which the editor alludes to in his preface, that tend to increase its convenience. The most important of these, in our view, is the addition of an index to each volume, besides the comprehensive triple index at the end of the series. But the general plan adopted at first has been wisely adhered to, and we can only say, in conclusion, that, with such careful editing and superb printing, it is a work that should receive the liberal patronage of the medical profession throughout the world.

